# Secretion systems and signal exchange between nitrogen-fixing rhizobia and legumes

**DOI:** 10.3389/fpls.2015.00491

**Published:** 2015-07-01

**Authors:** Matthew S. Nelson, Michael J. Sadowsky

**Affiliations:** BioTechnology Institute, Department of Soil, Water and Climate, University of Minnesota, Saint PaulMN, USA

**Keywords:** rhizobia, nodulation, symbiosis, signal exchange, type III secretion system, type IV secretion system, type VI secretion system, effector proteins

## Abstract

The formation of symbiotic nitrogen-fixing nodules on the roots and/or stem of leguminous plants involves a complex signal exchange between both partners. Since many microorganisms are present in the soil, legumes and rhizobia must recognize and initiate communication with each other to establish symbioses. This results in the formation of nodules. Rhizobia within nodules exchange fixed nitrogen for carbon from the legume. Symbiotic relationships can become non-beneficial if one partner ceases to provide support to the other. As a result, complex signal exchange mechanisms have evolved to ensure continued, beneficial symbioses. Proper recognition and signal exchange is also the basis for host specificity. Nodule formation always provides a fitness benefit to rhizobia, but does not always provide a fitness benefit to legumes. Therefore, legumes have evolved a mechanism to regulate the number of nodules that are formed, this is called autoregulation of nodulation. Sequencing of many different rhizobia have revealed the presence of several secretion systems - and the Type III, Type IV, and Type VI secretion systems are known to be used by pathogens to transport effector proteins. These secretion systems are also known to have an effect on host specificity and are a determinant of overall nodule number on legumes. This review focuses on signal exchange between rhizobia and legumes, particularly focusing on the role of secretion systems involved in nodule formation and host specificity.

## Introduction

Plants interact with many different types of microbes, and these associations can be pathogenic, mutualistic, or commensal in nature. The type of relationship between a specific microbe and plant can vary based on external factors, such as changes in environment, or due to intrinsic factors of both organisms. Both pathogenic and mutualistic interactions are dependent on communication between host and microbe and are primarily based on signal exchange (Tseng et al., 2009). The symbiotic relationship between rhizobia and legumes has long been a focus of study because of the nitrogen fixation that occurs during the symbiosis. This symbiosis requires the rhizobia to be in close physical proximity to the legume to allow for exchange of nutrients. Nitrogen is essential for all agricultural crops, but only legumes can access nitrogen from the atmosphere through symbiosis with rhizobia. Signal exchange between rhizobia and legumes has been studied as a potential process regulating symbiosis on non-legume plants and a mechanism by which to increase nitrogen fixation in legumes.

The symbiosis between legumes and rhizobia has evolved to incorporate many different levels of signal exchange, from initial contact to senescence. Two primary reasons for this signal exchange are to distinguish between symbionts and pathogens and to ensure mutualism through the exchange of carbon and fixed nitrogen. The line between symbiont and pathogen is not always clear, as both partners can have a fitness benefit to alter the relationship to their advantage. Symbiotic associations may shift from mutually beneficial to pathogenic or vice versa, such as in the case of the plant pathogen *Argobacterium*, having a common ancestral history with rhizobia. It has been suggested that rhizobia can be viewed as refined pathogens (Deakin and Broughton, 2009). The symbiotic relationship between rhizobia and legumes can easily turn pathogenic if the plants loses the ability to regulate the total number of nodules formed or the rhizobia form nodules that do not fix nitrogen – with the plant experiencing decreased fitness by providing too much carbon to the rhizobia (Herridge and Rose, 2000; Kiers et al., 2003). Co-evolution between rhizobia and legumes is more complex because of rhizobia selection can oscillate between pathogen and symbiont.

The evolutionary arms race between pathogens and plants has long been studied (Jones and Dangl, 2006). Pathogens develop new strategies for creating infections, such as evolving secretion systems to alter the host cell. In response, plants develop new strategies for detecting pathogens, such as microbe-associated molecular patterns (MAMPs), and R genes (Dodds and Rathjen, 2010). Sequencing of various rhizobial strains has shown the presence of secretion systems similar to those used by pathogens to transfer proteins into the hosts’ cytosol. These secretion systems include the Type III (T3SS), Type IV (T4SS), and Type VI secretion systems (T6SS; Fauvart and Michiels, 2008). The evolutionary presence of these secretion systems suggests that while rhizobia and legumes co-evolved a system allowing establishment and maintenance of a symbiosis, a relationship similar to a pathogen/plant interaction also co-evolved. This review focuses on legume–rhizobia signal exchange that occurs during nodule formation, plant mechanisms for limiting nodule number, and potential strategies used by rhizobia to overcome the plants ability to limit nodule number using the T3SS, T4SS, or T6SS.

## Signaling Exchange During Nodule Formation

Rhizobia are free-living, soil saprophytes, prior to symbiosis with plants in the family Leguminosae. Rhizobia, once inoculated into soil, can persist at low levels in the absence of a suitable host (Howieson, 1995). The plant initiates symbiosis by secreting flavonoids, which are detected by the rhizobia. Flavonoids vary by plant species and are only recognized by certain, yet specific, rhizobial species, offering the first level of symbiosis specificity (Hassan and Mathesius, 2012). The flavonoids diffuse across the membrane of the rhizobia and induce synthesis of the NodD protein to activate transcription of other genes involved in nodulation including nod factor (NF) production (Wang et al., 2012). NFs are a primary signal molecule produced by bacteria and detected by the plant to induce nodule organogenesis. Structurally NFs are lipochitooligosaccharides (LCOs) with a chitin oligomer backbone (Oldroyd and Downie, 2008). The *nodABC* genes encode for the proteins required to make the core NF structure and are conserved across all rhizobia species, except two *Aeschynomene*-infective species (Perret et al., 2000; Giraud et al., 2007). The NF core is then modified by species-specific proteins resulting in various substitutions on both the reducing and non-reducing end, including glycosylation and sulfation (Long, 1996). These substitutions are specific for each host legume and offer another level of symbiosis specificity (Dénarié et al., 1996; Long, 1996). Many surface polysaccharides are also involved in symbiosis specificity including lipopolysaccharides (LPSs), extracellular polysaccharides (EPSs), and capsular poylsaccharides (KPSs; Deakin and Broughton, 2009). The specific structure of LCOs is known to be important for recognition by host nod factor receptors (NFRs), which are receptor kinases containing lysin motifs (LysM; Radutoiu et al., 2007). Leucine rich repeat receptor-like kinases (LRR-RLKs) are also involved in NF perception and signaling, which results in nodule formation (Endre et al., 2002).

Root hair curling and crack entry are the two infection mechanisms used by rhizobia. Crack entry involves rhizobia entering through cracks at the lateral root bases or stems (Goormachtig et al., 2004). Root hair curling involves recognition of NFs, this recognition results in both calcium spiking and the curling of the root hair (Esseling et al., 2003). This is thought to involve a change in the plant cells’ polarity, resulting in a new growing direction of the root hair tip (Gage, 2004). The infection chamber enlarges and changes into a globular apoplastic space. Next, root tip growth in switched from radial to polar tip elongation (Fournier et al., 2015). The continued growth of the infection thread is dependent on NF specificity as well as EPS (Jones et al., 2007). Both the epidermis and the cortex recognize NFs, the epidermis regulates rhizobia infection and the root cortex is responsible for nodule formation (Oldroyd and Downie, 2008). Cortical cells develop into a nodule primordium. When the infection thread reaches the nodule primordium, the rhizobia enter into the inner cells and become encapsulated within a peri-bacteroid membrane (Oldroyd and Downie, 2008).

There are two main types of nodules, indeterminate and determinate, and this is determined by the legumes. For indeterminate nodules, cell division typically begins in the inner cortex (Ferguson et al., 2010). Indeterminate nodules maintain a persistent meristem and form distinct zones, including rhizobia invasion, active nitrogen fixation and senescence (Udvardi and Poole, 2013). These zones contain rhizobia in various developmental states with the proximal zone losing the ability to reproduce (Mergaert et al., 2006). Legumes belonging to the inverted repeat-lacking clade manipulate bacterial differentiation through secretion of cysteine-rich peptides, which induce membrane permeabilization, endoreduplication, and loss of independent viability (Mergaert et al., 2006; Van de Velde et al., 2010; Oldroyd et al., 2011). In contrast, cell division begins in the outer cortex for determinate nodules (Ferguson et al., 2010). Determinate nodules do not have a persistent meristem and form a homogenous group of rhizobia with full viability (Saeki, 2011). In mature nodules, plants exchange small carbon molecules for ammonia with the rhizobia. Another important aspect of symbiosis regulation is amino acid exchange and cycling between the plant and the rhizobia. During symbiosis some plants secrete branched chained amino acids, into the peribacteroid space, and in return the rhizobia secrete aspartate and, in some cases, alanine. Rhizobial biosynthesis of branched chained amino acids is shut down during symbiosis, preventing the use of ammonium by rhizobia and allowing the plant to incorporate ammonium into aspartate to produce asparagine (Lodwig et al., 2006; Prell et al., 2009).

After many weeks of plant growth, nodules begin to senescence, with a maximum lifespan well-short of that of the host plant (Puppo et al., 2005). Dark stress, water stress, defoliation, or addition of nitrate can initiate premature nodule senescence (González et al., 1998; Matamoros et al., 1999; Hernández-Jiménez et al., 2002). This suggest that the plant controls the duration of the symbiosis by being able to induce nodule senescence. These external factors are thought to lead to an increase in reactive oxygen species, which initiates senescence (Puppo et al., 2005). During nodule senescence, the host plant initiates plant cell death and some rhizobia not in the symbiosome survive this process and return to a saprotrophic state in the soil (Hernández-Jiménez et al., 2002).

## Plant Signaling Limits Nodule Number

The symbiotic relationship between rhizobia and legumes has the potential to become pathogenic if the plant loses the ability to regulate the total number of nodules or perceives the rhizobia as a pathogen. Rhizobia will generally initiate nodule formation because a symbiotic relationship always has a fitness benefit for the rhizobia. However, if the plant forms too many nodules then there is a negative effect on vegetative growth and yield (Herridge and Rose, 2000; Takahashi et al., 2003; Matsunami et al., 2004). Legumes use a process called autoregulation of nodulation (AON) to control nodule number by preventing new nodule formation (Mortier et al., 2012). The AON is thought to involve a root-derived signal being transported to the shoot, which induces a shoot-derived signal to be transported to the root – this inhibits nodule formation (Suzaki et al., 2015).

After nodule formation, the plant cell begins to produce CLV3/ESR-related (CLE) peptides. CLE peptides are thought to be the signal molecule transported from the roots to the shoot as part of the signaling pathway involved in AON (Reid et al., 2011, 2013). The CLE-RS2 is a post-translationally arabinosylated glycopeptide derived from the CLE domain, and if externally added CLE-RS2 sufficient to inhibit nodule formation (Okamoto et al., 2013). The CLE-peptides are recognized by LRR-RLKs (Krusell et al., 2002; Nishimura et al., 2002; Sasaki et al., 2014). These receptors then cause a signal cascade which results in cytokinins being transported from the shoot to the root, which could act as the shoot-derived signal to suppress nodule formation (Sasaki et al., 2014). In the *Lotus japonicas tml* mutant, shoot-applied cytokinin does not suppress nodule formation (Sasaki et al., 2014). This implies that TML acts downstream of cytokinins, and may act directly in the root cells to suppress nodulation. TML encodes a Klech repeat-containing F-box protein and has been hypothesized to target a protein for degradation which has a positive role in nodule formation (Takahara et al., 2013; Suzaki et al., 2015).

Autoregulation of nodulation signaling is a complex process involving numerous steps, some of which are still unknown. Disruption of AON at many different steps has been shown to results in a hyper-nodulation phenotype. This suggests that the AON signaling process could be potential targets for rhizobia to disrupt, in order to increase nodule formation. Inhibition of AON, could result in the symbiotic relationship between rhizobia and legumes becoming a pathogenic one (Herridge and Rose, 2000).

## Bacterial Secretion Systems

Bacteria use a wide variety of secretion systems to export proteins and other compounds across their membranes and cell walls. Interaction with the external environment is vital to bacterial survival, and many different transmembrane channels have evolved independently to fulfill this need (Wooldridge, 2009). There have been reports of up to many different secretion systems, but only the first seven have been significantly investigated (Tseng et al., 2009). These secretion systems have evolved independently, each containing a different set of core proteins. Each secretion system itself diverged into unique subfamilies based on different functions. The T1SS, T2SS, and T5SSs are thought to simply transport proteins and compounds outside of the cell. The T3SS, T4SS, and T6SSs contain subfamilies with the ability to transport effector proteins into the cytosol of eukaryotic cells (Wooldridge, 2009). This is important because it allows for the direct communication with, and modification of, the eukaryotic cytosol. These three secretion systems are well-understood for their role in pathogenesis as key factors in virulence and, in some cases, symbiosis.

## Rhizobia Secretion Systems

As discussed above, rhizobia enter into unique symbioses with eukaryotic cells, through the formation of relationship with legumes. Sequencing of rhizobia strains has shown that they typically contain multiple secretion systems. However, the presence of these systems in the bacterial genome does not mean they have a role in symbiosis. Rhizobia surface polysaccharides (LPS) have been known to suppress plant immune responses, but the T3SS and T4SS have also been speculated to have a role in suppressing the plant immune system (Masson-Boivin et al., 2009).

The T3SS and T4SS are each sub-divided into seven families based on function and protein homology (Wooldridge, 2009; Sugawara et al., 2013). The T3SS, T4SS, and T6SSs have been identified throughout various rhizobial genera and sequence homology shows similarity between known secretion systems used by bacterial pathogens. Specifically, sequence analysis of *Sinorhizobium* has shown that they can contain either the T3SS, T4SS or the T6SS, but typically only have one involved in symbiosis per strain (Sugawara et al., 2013). The T3SS, T4SS, and T6SS have all been shown to be involved in symbiosis and translocate effector proteins during symbiosis. These effector proteins could potentially have a function by promoting nodule formation, disrupting AON, or suppressing the plant’s immune response during invasion. In plant pathogens, the T3SS effectors have been shown to target and suppress the plant immune response (Macho and Zipfel, 2015). Deletion of a specific sub-family of the T3SS or the T4SS has been shown to reduce nodule number and affect host range specificity (Sugawara et al., 2013; Tampakaki, 2014). However, their role in symbiosis is still not very well-understood.

## Type III Secretion System

The T3SS is a structure composed of 20–27 different proteins, and this transporter is responsible for secretion of type III effector proteins (T3Es; Ghosh, 2004; Tampakaki, 2014). Approximately 50% of proteins involved in secretion system channel formation are conserved in most T3SSs (Ghosh, 2004). These proteins are generally found clustered in a 22–50 kb pathogenicity island (Tampakaki, 2014). The T3SS complex spans the bacterial inner and outer membrane as well as the hosts’ membranes and allows protein transport into the host. Regions flanking the pathogenicity island can contain genes that encode for effector proteins, but most effector genes are scattered throughout the genome (Lindeberg et al., 2008).

Many different variations of T3SS, with varying functions, are found throughout the kingdom of bacteria. In the literature, the T3SS is first grouped by species, and then grouped by homology. The genes encoding the rhizobial T3SSs are called rhc (*Rhizobium* conserved). The rhc are further subdivided into four families based on phylogenetic analyses, Rhc-1 to Rhc-4 (Gazi et al., 2012). Of these four families, only Rhc-I has been showed to be involved in symbiosis (Tampakaki, 2014). The functions of the other families are still unknown. The T3SS is among the best studied secretion systems in rhizobia due to the wide species distribution of Rhc-1 and its role in symbiosis.

### T3SS – Rhc-I Effect on Symbiosis

Early studies of the T3SS – Rhc-1 focused on knocking out the entire system through deletions or disruption of core genes. A diverse range of rhizobial species are known to contain a functional T3SS – Rhc-1 and are listed in **Table 1**. The influence of T3SSs on nodulation can vary from positive, in which nodulation is increased, to negative, in which nodulation is reduced. In *Sinorhizobium fredii* strain NGR234, the T3SS has both a positive and negative affect on multiple different legume species, but may also have a neutral phenotype, where nodulation is not affected, for example on *Vigna unguiculata* (Viprey et al., 1998; Skorpil et al., 2005; Kambara et al., 2009). Similarly, rhizobia with the T3SS – Rch-1 show host-dependent phenotypes in regard to nodulation efficiency. This could explain why the T3SS – Rch-1 is found in many genera of rhizobia, but is not ubiquitous at the strain level.

**Table 1:** Symbiotic effect of the T3SS – Rch-1 in rhizobia.

Strain of rhizobia with T3SS – Rch-1	Secreted proteins	Positive effect on symbiosis	Negative effect on symbiosis	Reference
*Rhizobium etli* CNPAF512	2	*Phaseolus vulgaris*	Unknown	Michiels et al. (1995), Fauvart and Michiels (2008)
*Bradyrhizobium elkanii* USDA61	8	*Macroptilium atropurpureum, Glycine max* ev. Clark, *G. max* cv. Enrei	*Vigna radiata* cv. KPS1, *G. max* cv. Hill	Okazaki et al. (2009), Okazaki et al. (2013)
*Mesorhizobium loti* MAFF303099	8	*Lotus glaber, Lotus japonicus, Lotus corniculatus* subsp. *frondsus, Lotus filicaulis*	*Leucaena leucocephala, Lotus halophilus, Lotus peregrinus* var. *carmeli, Lotus subbiflorus*	Hubber et al. (2004), Sánchez et al. (2009), Sánchez et al. (2012), Okazaki et al. (2010)
*Sinorhizobium fredii* NGR234	15	*Tephrosia vogelii, Flemingia congesta, Lablab purpureus*	*L. leucocoephala, Pachyrhizus tuberosus, Crotalaria juncea*	Viprey et al. (1998), Skorpil et al. (2005), Kambara et al. (2009), Kimbrel et al. (2013)
*S. fredii* HH103	8	*G. max* cv. Peking, Heinong 33, Kochi, and Williams, *Glycyrrhiza uralensis*	*Erythrina variegata*	Rodrigues et al. (2007), López-Baena et al. (2008)
*S. fredii* USDA207	13	Unknown	Unknown	Kimbrel et al. (2013)
*S. fredii* USDA257	13	*G. max* cv. Peking and Williams, *M. atropurpeum*	*G. max* cv. McCall, *E. variegata*	Krishnan et al. (2003), De Lyra et al. (2006), Kimbrel et al. (2013)
*Bradyrhizobium japonicum* USDA6	33	Unknown	Unknown	Kimbrel et al. (2013)
*B. japonicum* USDA110	36	*M. atropurpureum G. max* cv. Williams	*V. radiata* cv. KPS2	Krause et al. (2002), Wenzel et al. (2010), Kimbrel et al. (2013)
*B. japonicum* USDA122	31	Unknown	Unknown	Kimbrel et al. (2013)
*B. japonicum* USDA123	32	Unknown	Unknown	Kimbrel et al. (2013)
*B. japonicum* USDA124	33	Unknown	Unknown	Kimbrel et al. (2013)
*Cupriavidus taiwanensis* LMG19424	Unknown	Unknown	*L. leucocephala*	Saad et al. (2012)

^∗^Only strains with functional T3SS – Rch-1 with a known effect on symbiosis are listed. More strains have been sequenced that contain the T3SS – Rch-1, but these have not been experimentally tested for function (de Souza et al., 2012). The number of secreted proteins includes proteins identified through analysis of proteins found externally after induction of the T3SS, and proteins shown to be transported into the cytosol of *Arabidopsis*.

The horizontal transfer of the T3SS could be an important evolutionary driver toward symbiosis or pathogenesis between bacteria and plants. The pathogen *Ralstonia solanacearum* was shown to be unable to nodulate *Mimosa pudica* when the symbiotic plasmid of *Cupriavidus taiwanensis* was added, but was able to nodulate *M. pudica* if the T3SS was also deleted (Marchetti et al., 2010). This shows that the T3SS can prevent symbiosis. However, deleting the T3SS effector protein GALA7 prevented pathogenic infection of *Medicago truncatula* (Angot et al., 2006). This shows that the T3SS in *R. solanacearum* is required for pathogensis. In addition, *C. taiwanensis* was able to nodulate *Leucaena leucocephala* when the T3SS in *C. taiwanensis* was deleted (Saad et al., 2012). These examples show how the presence of the T3SS can restrict host range by preventing symbiosis, and could have a role in bacteria transitioning from a symbiont to a pathogen.

### Regulation of the T3SS – Rhc-1

Expression of the T3SS is induced by plant flavonoid recognition through production of the transcriptional activator TtsI (Viprey et al., 1998; Krause et al., 2002; Kobayashi et al., 2004). TtsI initiates transcription of the T3SS genes and effector proteins by binding to specific *cis*-elements, known as *tts* boxes (Wassem et al., 2008). The number and location of *tts* boxes varies between species and *Bradyrhizobium japonicum* USDA110 is known to have 52 different *tts* boxes. Proteins secreted by the T3SS are found downstream of *tts* boxes.

There is not a consensus motif for proteins secreted through the T3SS. However, the signal sequence is typically found in the first ∼15 amino acids, on the N-terminus, of translocated proteins (Ghosh, 2004). In addition not all gene transcription activated by *tts* boxes, are effector proteins translocated through the T3SS; some can have other roles in symbiosis such as the production of rhamnose-rich polysaccharides (Marie et al., 2004). These rhamnose-rich polysaccharides were shown to be surface LPSs, important in nodule formation, independent of the T3SS (Broughton et al., 2006). This suggests an interesting link between secretion systems and surface polysaccharides involved in nodule formation specificity.

### Proteins Secreted by the T3SS – Rhc-1

Early studies to identify proteins secreted through the T3SS focused on using flavonoids to induce expression in culture and compared the external proteins to those found in a T3SS mutant. However, these experiments did not show translocation into the host cytosol. This led to uncertainty as to whether an identified protein was an effector protein, acting inside the plant cell. A new, high-throughput technique was used to properly identify proteins that translocate through the T3SS as well as to identify effector proteins (Kimbrel et al., 2013). However, this technique did not test for effector translocation into legumes, but rather the proxy of translocation through *Pseudomonas syringae* pv. *tomato* DC3000 into *Arabidopsis* Col-O. The T3E candidates are fused to Δ79AvrRpt2, which induces a hypersensitive response (HR) in *Arabidopsis*. Using this technique on three different strains of *S. fredii* and *B. japonicum,* between 13 and 36 T3Es per strain were identified (Kimbrel et al., 2013). The T3Es can vary between species and strains, but members of the same species tend to use very similar effector proteins.

Proteins secreted by the T3SS can be separated into two categories – pilus forming and effectors. Proteins involved in pilus formation are secreted through the channel to assist in forming a channel through the plants cell wall or plasma membrane. NopA, NopB, and NopX are thought to be involved in the terminal formation of the T3SS, forming a pilus that penetrates the plant’s cell wall and plasma membrane (Lorio et al., 2004; Deakin et al., 2005; Saad et al., 2005, 2008). The other secreted proteins are thought to be effector proteins, but few of these proteins have a predicted function *in planta* (**Table 2**).

**Table 2:** Predicted functions of T3SS secreted proteins.

T3SS – Rch-1 secreted proteins	Strains containing homolog	Predicted function	Reference
NopA	*B. japonicum* USDA110, *M. loti* MAFF303099, *S. fredii* NGR234, *S. fredii* HH103, *S. fredii* USDA257	Part of the T3SS extracellular pilus which spans the plants cell wall	Deakin et al. (2005), Saad et al. (2008)
NopB	*B. japonicum* USDA110, *M. loti* MAFF303099, *S. fredii* NGR234, *S. fredii* HH103, *S. fredii* USDA257	Part of the T3SS extracellular pilus which spans the plants cell wall	Saad et al. (2005), Saad et al. (2008)
NopD	*S. fredii* HH103	Homology to a predicted C48 cysteine peptidase	Hubber et al. (2004), Rodrigues et al. (2007)
NopL	*B. japonicum* USDA110, *S. fredii* NGR234, *S. fredii* HH103, *S. fredii* USDA257	Suppresses cell death induced by mitogen-activated protein kinase (MAPK)	Zhang et al. (2011)
NopM	*B. japonicum* USDA110, *S. fredii* NGR234, *S. fredii* HH103	E3 ubiquitin ligase, thought to be involved in protein–protein interactions	Rodrigues et al. (2007), Xin et al. (2012)
NopP	*S. fredii* NGR234, *R. etli* CNPAF512, *S. fredii* HH103, *S. fredii* USDA257	Phosphorylated by plant kinases	Skorpil et al. (2005)
NopT	*S. fredii* NGR234	Cysteine protease	Fotiadis et al. (2012)
NopX	*M. loti* MAFF303099, *S. fredii* NGR234, *S. fredii* HH103, *S. fredii* USDA257	Terminal part of the T3SS extracellular pilus which spans the plants cell wall	Saad et al. (2008)
Mlr6361	*M. loti* MAFF303099	Shikimate kinase	Sánchez et al. (2009)

^∗^Subset of known proteins secreted by the T3SS – Rch-1. Only proteins with a predicted function or that have been experimentally tested are listed. None of the proteins have been tested in legumes, but some have been tested *in planta* in *Nicotiana tabacum.*

As shown in **Table 1**, deleting the T3SS can have a positive or negative effect on symbiosis. The T3SS is simply the means of transport for effector proteins. Deleting the T3SS prevents effector protein transport. These effector proteins play key roles in symbiosis. Despite having a known effect on symbiosis, none of these effector proteins has been expressed in legumes. Only the effectors NopL, NopT, and NopM have all been expressed in eukaryotic cells. NopL was first shown to be phosphorylated by plant kinases (Bartsev et al., 2003). Next, NopL was shown to interfere with mitogen-activated protein kinase (MAPK) signaling in *Nicotiana tabacum*. MAPK signaling is involved pathogen recognition in both basal plant defense and R-mediated resistance (Pedley and Martin, 2005). Part of the plant defensive response is the induction of HR. The plant pathogen *P. syringae* uses effector proteins AvrPto and AvrPtoB to interrupt MAPK signaling by degrading the plant protein FLS2 (Göhre et al., 2008; Shan et al., 2008). Overexpression of MAPK signaling in plants induces HR to prevent pathogen infections. NopL was shown to suppress cell death induced by the overexpression of MAPK signaling (Zhang et al., 2011). NopT when expressed in *N. tabacum* or *Arabidopsis thaliana* elicited a strong HR response and necrotic symptoms. The authors did suggest that it could function as a protease and had similarity to the effector family YopT – AvrPphB (Dai et al., 2008). AvrPphB is an effector in *P. syringae* and functions as an autoprotease, cleaving itself to expose a myristolation site (Puri et al., 1997; Shao et al., 2002). The addition of myristoyl groups after cleavage, target AvrPphB to the cell membrane (Nimchuk et al., 2000). NopT has been shown to have cysteine protease activity and may use autoproteolysis for target to cell membranes, but its role is still uncertain (Fotiadis et al., 2012). NopM was shown to possess E3 ubiquitin ligase activity. Furthermore, when this ability was lost through a point mutation, the positive effects on nodule formation were also lost (Xin et al., 2012).

Even though the function of many specific proteins has not been determined, the accumulated effect of the T3SS effector proteins can be determined through deletion of the entire secretion system. *Bradyrhizobium elkanii,* containing the T3SS, but not the T3SS mutant, was shown to increase the transcription of two genes in the roots of a soybean line deficient in NF recognition (Okazaki et al., 2013). These genes, *ENOD40* and *NIN*, are involved in early nodulation regulation. This suggests that the T3SS effector proteins may be involved in up-regulating host genes involved in nodule formation. Further research is needed to more completely understand how these individual effectors are functioning *in planta*.

## Type IV Secretion System

The T4SS-b is functionally similar to the T3SS-Rch-1 and is also involved in protein translocation, but has a separate evolutionary origin. The T4SS is generally sub-divided into three families based on function, including conjugation, DNA uptake and release, and protein translocation (Cascales and Christie, 2003). These three families can use similar core proteins to form the main channel and may share sequence similarity. Properly identifying which sub-family is present in a specific strain is key. In rhizobia, the T4SS-b shares strong homology to the VirB/VirD4 subunits found in *Agrobacterium*. The core structure consists of 12 proteins, VirB1-B11 and VirD4. The T4SS-b, in *Agrobacterium tumefaciens*, is used for translocation of both T-DNA and effector proteins (Kuldau et al., 1990; Zupan and Zambryski, 1995). The function of the T4SS-b is well-understood because of its role in plant transformation. *Agrobacterium* and rhizobia are closely related, and understanding of the T4SS-b in *Agrobacterium* has been leveraged to better understand the T4SS-b in rhizobia.

### T4SS-b Effect on Symbiosis

Unlike the T3SS, there is a paucity of information regarding the role of the T4SS in symbiosis. A functional T4SS-b has only been identified in three different species (**Table 3**). Similar to the T3SS, the T4SS-b can have both a positive or negative effect on symbiosis. In *Mesorhizobium loti* R7A, nodulation on *Lotus corniculatus* reduced, but not completely lost, when the T4SS-b was partially deleted. This same deletion allowed *M. loti* R7A to gain the ability to form nodules on *L. leucocephala* (Hubber et al., 2004). Deleting the T4SS-b in *Sinorhizobium meliloti* KH46c resulted in approximately a 50% decrease in nodule number on *M. truncatula* A17, but did not have a significant effect on *M. truncatula* F83005-5 (Sugawara et al., 2013). This dual positive and negative selection could explain why only 9 of 33 *S. meliloti* and 11 of 13 *S. medicae* strains were found to contain the T4SS-b (Sugawara et al., 2013).

**Table 3:** Symbiotic effect of the T4SS-b.

Strain of rhizobia with T4SS – B	Secreted proteins	Positive effect on symbiosis	Negative effect on symbiosis	Reference
*M. loti* R7A	2	*L. corniculatus*	*L. leucocephala*	Hubber et al. (2007)
*S. meliloti* KH35c	Unknown	*M. truncatula* A17, *M. tricycla*	Unknown	Sugawara et al. (2013)
*S. medicae* M2	Unknown	*M. truncatula* A17	Unknown	Sugawara et al. (2013)

^∗^Only strains with a functional T4SS-b are listed. More strains containing a T4SS-b have been sequenced, but not experimentally tested for function (Sugawara et al., 2013). Both secreted proteins in *M. loti* R7A, have been shown to be translocated into *Arabidopsis* (Hubber et al., 2004).

### Regulation of the T4SS-b

Transcription of the T4SS is controlled by a two-component response regulator VirA/VirG (Stachel and Zambryski, 1986). VirA is a membrane bound kinase that phosphorylates VirG in response to external factors (Hansen et al., 1994). In contrast, VirG is a transcriptional activator that binds to *vir* boxes. In *Rhizobium* these regulators are induced by flavonoids that activate VirG (Hubber et al., 2007). Unlike the T3SS effectors, which can be present throughout the genome, T4SS tend to be near VirG (Vergunst et al., 2000; Tampakaki, 2014). Research in *A. tumefaciens* has identified a sequence motif, a positive charged C-terminus, present on effector proteins needed for translocation (Vergunst et al., 2005). This same sequence motif is also present on the only two effector proteins identified, Msi059 and Msi061, both in *M. loti* R7A (Hubber et al., 2004). VirD4 interacts with the positive charge signal sequence to transport the protein through the channel (Vergunst et al., 2005). VirD4, and the requirement of a more specific signal sequence, could result in more specificity in protein transport.

### Proteins Secreted by the T4SS-b

Thus far, only two proteins have been shown to transport through the T4SS-b, Msi059, and Msi061 in *M. loti* R7A. The Msi059 showed partial protein sequence similarity to a C48 cysteine peptidase. Interestingly, the NopD T3E in *S. fredii* HH103 also was a predicted C48 cysteine peptidase (Rodrigues et al., 2007). The C48 cysteine peptidase family contains the protein XopD, a T3E from the plant pathogen *Xanthomonas campestris* (Hotson et al., 2003). XopD encodes an active cysteine protease, and functions *in planta* to target SUMO-conjugated proteins (Hotson et al., 2003). This interferes with the plant’s ability to regulate the expression of specific proteins. Msi061 has shared protein similarity with *A. tumefaciens* effector VirF. The VirF interacts with the host Skp1 to facilitate protein degradation of effector proteins VirE2 and Vip1 to unbind the T-DNA after into the host cell (Schrammeijer et al., 2001; Tzfira et al., 2004). Skp1 is a core component of the E3 ubiquitin ligase, which mediates protein degradation (Schrammeijer et al., 2001). The precise activity of Msi059 and Msi061 are still unknown, but current evidence suggests a role in changing protein expression levels *in planta*.

## Type VI Secretion System

The T6SS is among the least researched secretion system involved in protein translocation. The T6SS is known to contain different subfamilies, but the sub-families and their functions have yet to be clearly defined. The number of proteins involved in forming the core structure seem to vary and there is no known secretion signal for protein transport (Bingle et al., 2008). Additionally, how T6SS expression is regulated is unknown. Still, the T6SS is thought to play an important role in the virulence of multiple pathogens, like *Burkholderia mallei* (Schell et al., 2007).

### T6SS Effect on Symbiosis

The sequence for the T6SS has been found in five different species of rhizobia, *R. leguminosarum*, *B. japonicum*, *M. loti, S. saheli,* and *S. fredii* (Bladergroen et al., 2003; Bingle et al., 2008; Sugawara et al., 2013). However, a functional T6SS, with an effect on symbiosis, has only been shown in *R. leguminosarum.* In this bacterium a negative effect on symbiosis was observed, where the T6SS prevented nodulation on *Pisum sativum* cv. Rondo (Bladergroen et al., 2003). A single protein was identified that is secreted through the T6SS. Sequencing of the first 50 amino acids suggested a role in ribose transport (Bladergroen et al., 2003). The effect that ribose transport has on symbiosis is unclear. More strains containing the T6SS have been identified, but not experimentally tested for function (Bingle et al., 2008; Sugawara et al., 2013).

## Example of Effector Involvement in Symbiosis

Most studies have focused on deleting specific genes in the core structure, instead of the effector proteins, and observing the overall phenotypic change. This is likely due to the fact that the core genes, unlike effectors, do not vary between species. Additionally, the phenotypic effect(s) of a single effector knockout might be small, again with some strains containing 36 different T3Es. One of the most well-characterized examples of the how the T3SS functions is in *S. fredii* strain USDA257. In this case *S. fredii* USDA257 is both a pathogen and a symbiont.

Legumes limit nodule number, and one mechanism used is to abort nodule formation, through a process similar to HR (Vasse et al., 1993). The *S. fredii* USDA257 strain contains NopL, which suppresses cell death through preventing MAPK signaling from inducing HR and cell death (Bartsev et al., 2004; Zhang et al., 2011). This would, in theory, increase the total number of nodules formed. Soybeans have evolved an R gene, Rfg1, capable of detecting T3Es from *S. fredii* USDA257 (Yang et al., 2010). Rfg1 encodes a TIR-NBS-LRR disease resistance protein, which are known to recognize pathogen effectors to induce disease resistant (Belkhadir et al., 2004). In soybean lines expressing Rfg1, the plant prevents nodulation by *S. fredii* USDA257, but not in the T3SS knockout mutant (Trese, 1995; Yang et al., 2010). In addition *S. fredii* USDA257 formed almost twice as many nodules on the soybean lines without the Rfg1 and the recessive rj2 genes as did the T3SS knockout mutant, on three different soybean lines (Yang et al., 2010). Taken together, the T3SS, including NopL, can increase nodulation in soybean. Recognition of the T3Es, by Rfg1, results in complete prevention of nodulation. NopL restricts the plant’s ability to prevent infection and nodule formation, and rhizobia become partially pathogenic through using this strategy. The specific protein which is recognized by Rfg1, either directly or indirectly, is still not known. Though this is just one example, it is consistent with observations from other studies showing both the positive and negative effects of the T3SS as listed in **Table 1**. This dual selection also explains why the T3SS is not found in all strains of Rhizobia.

## Proposed Model

Most of these studies were done by deleting the entire secretion system, versus knocking out only specific effector proteins. Secretion systems are not found in all strains for any species of rhizobia. Typically, if the T3SS or T4SS has a positive effect on nodulation, then deletion of the T3SS results in ∼40–60% reduction in nodule number. This shows that secretion systems are not essential for effective nodulation. If the T3SS has a negative effect on nodulation, then knocking out the T3SS or T4SS results in a gain of function phenotype, where the strain is now able for form nodules on a host genotype that it was previously unable to nodulate effectively. This shows that secretion systems restrict host range. Taken together, the evidence suggests that effector proteins may act in a pathogenic manner. The function of most effector proteins are not known. Many are predicted to modify *in planta* protein levels, and NopL was shown to suppress defense responses. This suggest that rhizobial effector proteins act in a pathogenic manner, similar to the function of other known bacterial effector proteins (Shames and Finlay, 2012).

**FIGURE 1:**
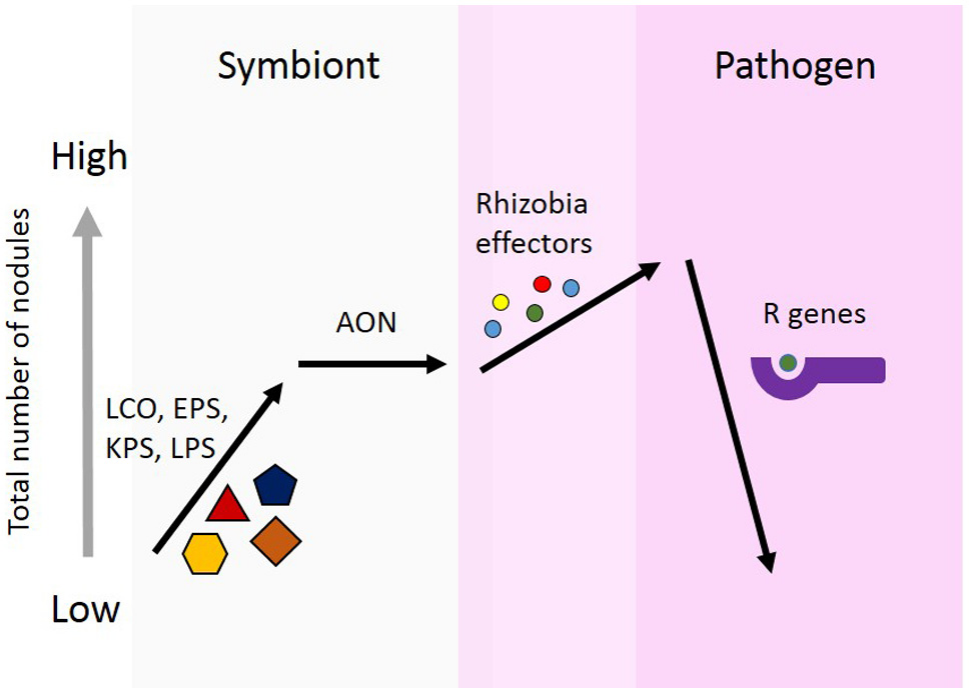
**Proposed model for the role of effector proteins in symbiosis.** Rhizobia secrete nod factors, which are lipochitooligosaccharides (LCOs), and are important for nodule formation and host specificity. Surface polysaccharides are also known to be involved in determining specificity for nodule formation. These include, extracellular polysaccharides (EPSs), capsular polysaccharides (KPSs), and lipopolysaccharides (LPSs). Legumes limit the total number of nodules formed using autoregulation of nodulation (AON). Rhizobia use effector proteins, similar to pathogens, to alter plant cells to facilitate increased nodule formation. Effectors alter the symbiotic state toward pathogenesis. In response, plants can develop R genes capable of recognizing the presence of these effector protein, either directly or indirectly. Effector recognition results in the plant initiating a defense response and preventing nodule formation.

The model we propose here (**Figure 1**), is to demonstrate three points regarding effector proteins: (1) the role of effector proteins is strictly pathogenic, and not involved in symbiosis communication between the rhizobia and host; (2) the role of effector proteins may lead to an increase nodule number. AON is the plants system for regulating nodule number. The mechanism of action for individual effector proteins will differ, but the unifying aspect is the increase in nodule number. This increase could be achieved through forming additional nodules or the prevention of nodule senescence; and (3) plants use R genes to recognize effector proteins. This recognition results in host defense responses, which can prevent nodulation. This serves to establish a host range for rhizobial strains possessing effector proteins which are recognized by the host.

## Conclusion

The T3SS, T4SS, and T6SS all play an important role in nodule formation in the symbiosis between rhizobia and legumes. Many studies have shown that these secretion systems have an effect on host range. NFs and surface polysaccharides are also known to effect symbiotic host range. These factors are important for host recognition of a symbiont versus a pathogen and facilitate infection for nodule formation. However, pathogens use effector proteins during invasion to promote virulence, and these effectors have an effect on the pathogens host range. Thus, other factors besides host range have to be used to determine the role of secretion systems in rhizobia/plant interaction. The T3SS, T4SS, and T6SS are all known to transport effector proteins. The predicted function of these proteins *in planta*, plus identifying R genes which respond to the T3SS or its effectors, strongly suggest that these secretion systems are acting in a pathogenic manner.

These secretion systems function to transport proteins from rhizobia into the plant cytosol. Once in the cytosol, they act to either increase nodulation or result in decreased nodulation through plant defense recognition. Specific changes *in planta* are not yet known. Identifying how rhizobia use effector protein could have an important agricultural application. Rhizobia may be using these proteins to suppress or prevent AON, and manipulation of this regulation may lead to the development of new strategies for increasing nodule formation. These effector proteins still have not been expressed *in planta*, in legumes, and thus their functions remain unclear. Although several hypotheses have been postulated, the role of T3SS and T4SS are still not fully understood and warrant further research.

## Conflict of Interest Statement

The authors declare that the research was conducted in the absence of any commercial or financial relationships that could be construed as a potential conflict of interest.
